# *In ovo* carvacrol enriched inflammatory and T-cell transcriptional responses to *Escherichia coli* LPS in broiler chickens

**DOI:** 10.3389/fimmu.2026.1761404

**Published:** 2026-03-03

**Authors:** Mila M. Y. Meijer, Henry van den Brand, Shahram Niknafs, Eugeni Roura

**Affiliations:** 1 Queensland Alliance for Agriculture and Food Innovation (QAAFI), The University of Queensland, Brisbane, QLD, Australia; 2 Adaptation Physiology Group, Department of Animal Sciences, Wageningen University and Research, Wageningen, Netherlands

**Keywords:** broiler chicken, broiler embryo, carvacrol, immunomodulation, *in ovo*, LPS, transcriptomics

## Abstract

**Introduction:**

Enhancing immune responsiveness against pathogens is crucial for maintaining health. In broiler chickens, the *in ovo* delivery of carvacrol, a phenolic compound found in oregano and thyme, has shown promising immunomodulatory activity. This study investigated the hypothesis that the *in ovo* delivery of carvacrol would regulate the immune response against *Escherichia coli* lipopolysaccharide (LPS) in broiler chickens.

**Methods:**

*In ovo* carvacrol (injected at embryonic day 17.5) and/or *E. coli* LPS challenge (repeated intraperitoneal injections at post-hatching d7 and d14) were tested in a 2 × 2 factorial arrangement, resulting in four groups: 1) *in ovo* saline without challenge (saline), 2) *in ovo* saline with LPS challenge, 3) *in ovo* carvacrol without challenge, and 4) *in ovo* carvacrol with LPS challenge. Performance parameters were collected, and relative organ weights were measured at d14. Splenic samples were collected 6 h after the second challenge (d14) and used for transcriptomic analyses.

**Results and Discussion:**

The LPS challenge resulted in lower chicken weight gain and higher relative spleen weight. The *in ovo* delivery of carvacrol had no impact on these measures. The transcriptomic comparisons for the effects of LPS challenge identified 786 differentially expressed genes (DEGs) between groups 2 and 1 (without *in ovo* carvacrol) and 1,832 DEGs between groups 4 and 3 (with *in ovo* carvacrol) [false discovery rate (FDR)< 0.05, −0.5 > logFC > 0.5], primarily enriching inflammatory pathways (p< 0.01). The transcriptomic comparisons for the effects of *in ovo* carvacrol identified 89 DEGs between groups 3 and 1 and 720 DEGs between 4 and 2 (p< 0.05, −0.5 > logFC > 0.5). Functional analyses (4 vs. 2) showed that the *in ovo* delivery of carvacrol enriched pathways that can be related to T-cell activation at 6 h after LPS challenge, mainly influenced by the upregulation of DEGs encoding for cytokines and chemokines (*p*< 0.01). Overall, these findings suggest that the *in ovo* delivery of carvacrol may modulate immune responses toward an *E. coli* LPS challenge, specifically by upregulating the gene expression of cytokines, chemokines, and their receptors, persisting until at least 14 days post-hatching.

## 1 Introduction

After hatching, broiler chickens are particularly vulnerable to pathogenic infections and other environmental stressors that can adversely affect their growth, health, and welfare (1, 2). Genetic selection strategies have emphasised growth at the expense of immune function, often resulting in compromised immune responsiveness (3, 4). Since the immune system plays a crucial role in the defence against pathogens, modulating immune responsiveness without negatively impacting performance is a key objective in poultry management, particularly in early life stages when broiler chickens are more susceptible to diseases due to an immature immune system (5).

A promising approach for achieving these goals involves the use of natural compounds. Carvacrol, a phenolic compound found in essential oils such as oregano and thyme, is known for its antimicrobial and short-term immunomodulatory properties. Previous studies have demonstrated the effectiveness of dietary carvacrol supplementation in modulating immune responses in broiler chickens post-hatching (6, 7). The effects of carvacrol through *in ovo* delivery—injecting substances into the fertile egg during embryonic development—present an opportunity to enhance immune responses already at an earlier developmental stage (8–10). Our previous research has demonstrated that, even in the absence of an inflammatory stimulus, the immunomodulatory effects of the *in ovo* delivery of carvacrol persisted until at least d14 post-hatching, as shown by changes in immune-related gene expression in the jejunum and bursa of Fabricius, along with morphological changes in the bursa of Fabricius (11). However, it remains unclear whether the *in ovo* delivery of carvacrol affects immune pathways in the presence of an inflammatory stimulus and which physiological mechanisms are involved.

The aim of this research was to determine if the *in ovo* immunomodulatory effect of carvacrol extended to later stages (post-hatched) of development (d14), particularly in response to an *Escherichia coli* lipopolysaccharide (LPS) challenge. Although carvacrol has previously been described as anti-inflammatory, its effects following *in ovo* delivery and during a later immune challenge are unclear. We therefore hypothesised that *in ovo* carvacrol would regulate immune-related splenic gene expression during an LPS challenge, without predefining the direction of this response.

## 2 Materials and methods

### 2.1 Experimental design

To test the effects of the *in ovo* delivery of carvacrol during an immune challenge, a post-hatching intraperitoneal injection of LPS program was used as an inflammatory challenge model.

**Figure 1:**
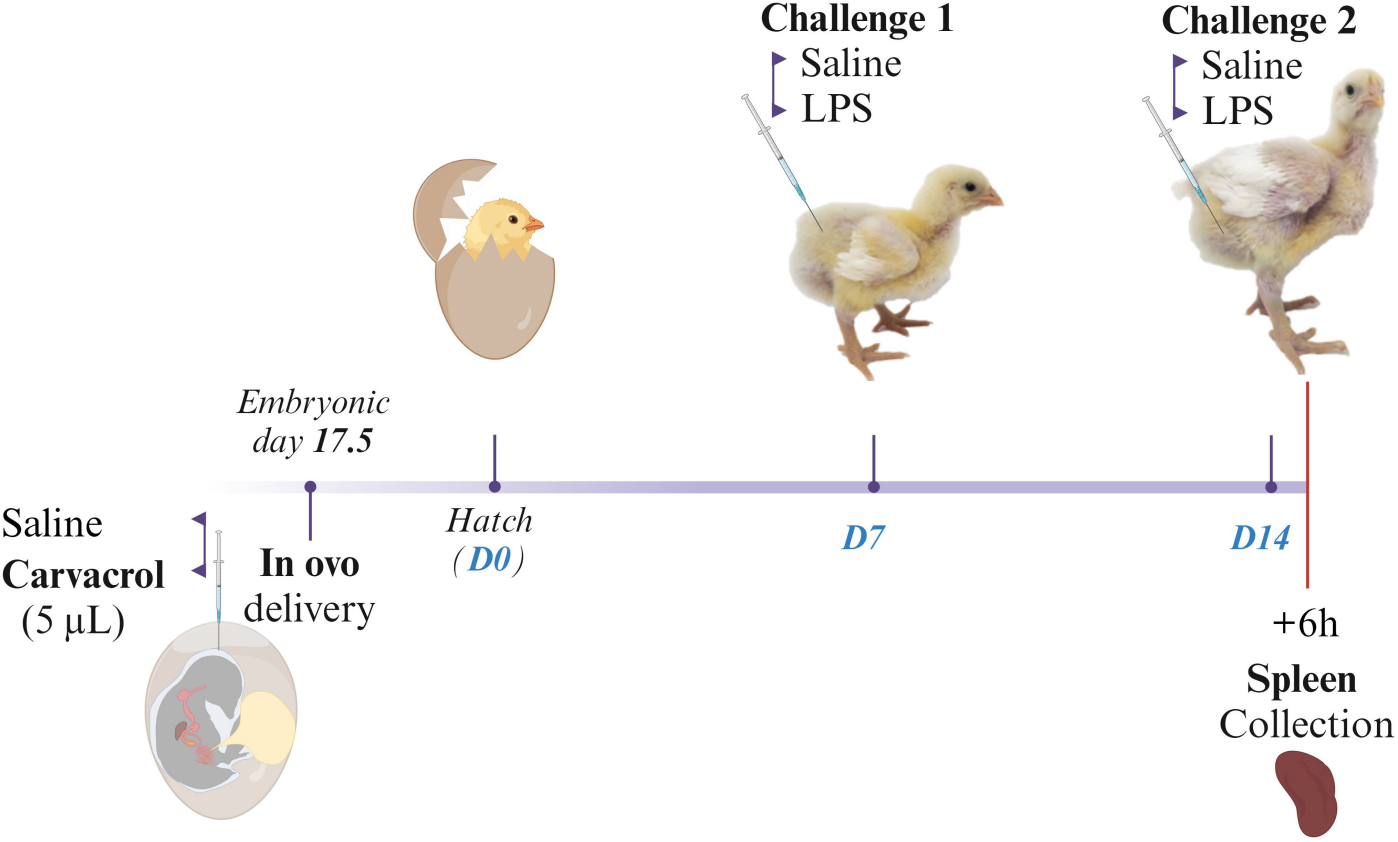
Timeline of procedures and sample collection. Broiler embryos were injected with a saline or carvacrol solution at embryonic d17.5. After hatching, chickens were challenged with saline or *Escherichia coli* lipopolysaccharide (LPS) at d7 and d14. Splenic samples were collected 6 h after the second challenge at d14. Created with BioRender.com.

This experiment followed a 2 × 2 factorial arrangement, including two *in ovo* delivery groups (a control group receiving saline solution and a treatment group receiving carvacrol) and two immune challenge treatments (saline or LPS) (**Figure 1**). This resulted in the following treatments: *in ovo* saline with no immune challenge (CTRCTR), *in ovo* saline with LPS challenge (CTRLPS), *in ovo* carvacrol with no immune challenge (CARCTR), and *in ovo* carvacrol with LPS challenge (CARLPS). The *in ovo* delivery was performed on embryonic day (E) 17.5 into the amniotic fluid of fertile broiler eggs. Subsequently, at d7 and d14 post-hatching, chickens received intraperitoneal injections of either saline or LPS. Splenic samples were collected at d14, 6 h after the second challenge.

### 2.2 Eggs, chickens, and housing

A total of 160 Ross 308 eggs from a single breeder flock (32 weeks of age), with an average egg weight of 56 g (SD = 1.6 g), were acquired from a commercial hatchery (Woodlands, Beerwah, QLD, Australia) and transported to the University of Queensland experimental chicken hatchery (St Lucia, Queensland, Australia). Eggs were incubated in a six-level setter (Ova-Easy 580 Advance Series II, Brinsea, FL, USA). Out of six levels, the top five were used with two trays per level, totalling 10 trays. The moment the eggs were placed in the preheated setter was defined as the start of incubation. Incubation in the setter lasted 17.5 days (until *in ovo* injection), maintaining a set incubator temperature of 37.8°C, relative humidity of 57%, and a turning interval of 60 min at a 90° angle. On E17.5, fertile eggs were injected (see *in ovo injection*) and transferred to a hatcher (Greatlander 6BH Six Basket Hatcher, Australia). In the hatcher, six baskets were stacked vertically, each basket divided into 32 single-egg windowed compartments. To ensure a balanced distribution of both saline and carvacrol-injected eggs across baskets, each egg was assigned to a compartment following a predefined randomised design. The lowest basket was not used. In the hatcher, the incubation process continued until hatching, with hatcher temperature and humidity levels maintained at 37.8°C and 70%, respectively. Starting at E20 (480 h after the start of incubation), the hatcher was monitored every 12 h for hatched chickens. After hatching, the following measurements were performed: hatchability (% of fertile eggs), hatched body weight (BW), navel quality [as described by Molenaar et al. (12)] and chicken quality (1 = good, 2 = deformed and to be euthanised, and 3 = dead in basket). Subsequently, 60 first-grade chickens (chickens with no observed deformities) per *in ovo* treatment were moved to cage brooder units (Cimuka, Turkey) for rearing until d14. Chickens were divided into two immune-challenged treatments (saline or LPS) per *in ovo* treatment, resulting in four different treatment combinations with 30 chickens each. A total of five brooder units were used, each consisting of five stacked cages (90 × 44 × 24 cm), leaving the lowest cage empty. Chickens were randomly allocated to five cages (n = 5) per treatment combination, with each cage housing six chickens of the same treatment. *Ad libitum* access to water and feed was provided, with cage temperature initially maintained at 30 °C–32°C for the first 3 days, followed by a decrease to 28°C at d4, which was kept constant until d14. Lighting conditions followed a schedule of 23 h of light and 1 h of darkness for the first 3 days, transitioning to six continuous hours of darkness (11 PM–5 AM) for the remainder of the study. On d7, 10 chickens per treatment group (two from each cage) were euthanised by cervical dislocation for sample collection. On d14, a further 10 chickens per treatment group were euthanised. At the end of the trial (d14), all remaining birds were euthanised by cervical dislocation.

### 2.3 *In ovo* injection

At E17.5, eggs were candled, and infertile eggs were removed. Fertile eggs were injected with either saline or carvacrol solutions via *in ovo* injection (n = 80 per treatment). Prior to injection, eggs were sterilised using a 70% ethanol swab. Using a multi-purpose rotary tool (Ryobi EHT150, Ryobi, Hiroshima, Japan) equipped with an arrow-shaped insert (Dremel High-Speed Cutter 6.4 mm, Dremel, Mount Prospect, IL, USA), a hole was carefully drilled into the blunt side of the eggshell, ensuring that the egg membranes remained intact. Injection into the amniotic fluid was performed using a 23G 1¼ (32 mm) precision needle. Subsequently, the puncture sites were sealed using beeswax, and the eggs were transferred to the hatcher.

For injection, either 1 mL of 0.9% sterile saline solution or 5 µL of carvacrol (Sigma-Aldrich, St. Louis, MO, USA; CAS: 499-75-2) in 1 mL of 0.9% sterile saline solution was administered. The carvacrol solution consisted of 2 mL of carvacrol to which 2 mL of the non-ionic surfactant polysorbate 80 (Tween^®^80, Sigma-Aldrich, St. Louis, MO, USA; CAS: 9005-65-6) was added for solubilisation. This mixture was blended by gently pipetting for 1 min. Following this, 2 mL of 0.9% sterile saline solution was added and mixed thoroughly with the carvacrol–polysorbate 80 solution by pipetting. This process of addition and mixing was repeated three more times, which resulted in a total volume of 12 mL. To adjust to a final volume of 40 mL, 28 mL of sterile saline was added and mixed by shaking. This 5% carvacrol stock solution was then diluted 10-fold with 0.9% sterile saline to produce working solutions containing 0.5% carvacrol.

### 2.4 Challenge, sampling, and measurements

On d7 post-hatch, individual BW and feed intake (FI) per cage between d0 and d7 were recorded. All chickens were then intraperitoneally injected using 30 G × 8 mm hypodermic needles, with either 200 µL of 0.9% sterile saline solution (NaCl 0.9% in water, Baxter, Deerfield, IL, USA; CAS: 7647-14-5) or 2 mg/kg BW LPS (from *E. coli* O55:B5, Sigma-Aldrich, CAS: 93572-42-0) in sterile saline. Dosages were calculated based on the Ross 308 target BW at the time of injection (Ross 308 performance objectives 2022). This process was repeated at d14, again with either sterile saline or 2 mg/kg BW LPS but using a larger volume (500 µL). Feed conversion ratio (FCR) was calculated using body weight gain (BWG) and FI from d0 to d14 and from d7 to d14. Before sample collection (6 h post-challenge), all remaining chickens were euthanised by cervical dislocation.

### 2.5 Statistical analyses

Performance and organ weight data were analysed using the statistical software package SAS 9.4 (SAS Institute Inc., Cary, NC, USA).

The individual chicken was considered the experimental unit for organ weights. For BW before and after the challenge, BWG, FI, and FCR, the cage was the experimental unit. Organ weights were expressed as a percentage of BW at the time of organ collection.

A generalised linear mixed model (Proc Mixed) was used for BW, organ percentages, BWG, FI, and FCR. Model assumptions were validated for both the means and residuals.

For all performance data, the basic model used (Equation 1) was as follows:

(1) $${\text{Y}}_{\text{i}}=\text{μ}+{\text{Treatment}}_{\text{i}}+{\text{Challenge}}_{\text{j}}+\text{Treatment}\times {\text{Challenge}}_{\text{ij}}+{\text{e}}_{\text{ij}}$$

where Y = the dependent variable, µ = the overall mean, Treatment_i_ = *in ovo* treatment (i = saline or carvacrol injected), Challenge_j_ = j = saline or LPS, Treatment × Challenge_ij_ = the interaction between the Treatment and Challenge, and e_ij_ = the residual error term.

Data are shown as LSmeans ± pooled SEM, with multiple comparisons between treatments adjusted using Tukey’s method. Differences between treatments were considered significant at *p* ≤ 0.05.

### 2.6 Transcriptomic analysis

#### 2.6.1 Tissue sampling and RNA extraction

Six hours after the second challenge (d14), 10 chickens per treatment group (two from each cage) were selected based on the average weight per cage. BW was recorded, and chickens were euthanised by cervical dislocation. Liver, bursa, and spleen weights were recorded and expressed as a percentage of total BW. Approximately 5 mm^3^ of spleen tissue was collected, manually rinsed with phosphate buffered saline (PBS) for 10 s, and stored in RNAlater (Sigma-Aldrich, St. Louis, MO, USA) at −80 °C until analysis. Total RNA was isolated from the splenic tissues (n = 5 per treatment, one chicken from each cage) using the RNeasy Mini kit (Qiagen, Hilden, Germany) following the manufacturer’s instructions. In brief, 20 to 30 mg of splenic tissue was homogenised in a QIAzol lysis reagent (Qiagen, Hilden, Germany) using a TissueRuptor (Qiagen, Hilden, Germany). Subsequently, a gDNA eliminator solution (Qiagen, Hilden, Germany) was used to remove genomic DNA. Then, multiple washing steps were performed with 70% ethanol and washing buffers using RNeasy spin columns and a micro-centrifuge according to the manufacturer’s instructions. Purified RNA was diluted with RNase-free water and stored at −80 °C. The evaluation of RNA samples (quality and quantity) was conducted using an Agilent 2100 Bioanalyzer (Agilent Technologies, Santa Clara, CA, USA). All samples had a quality score of RNA integrity number (RIN) between 9.6 and 10.0 and were used for sequencing.

#### 2.6.2 Library preparation and RNA sequencing

The Australian Genome Research Facility (AGRF Ltd., Melbourne, VIC, Australia) performed both library preparation and RNA sequencing, which followed the requirements of ISO17025 as outlined by the National Association of Testing Authorities (NATA) of Australia. In brief, using the Illumina NovaSeq X Plus platform, sequencing was conducted on all 20 poly-A RNA-stranded samples, resulting in an average of 47.5 million paired-end sequence reads per sample (average of 14.4 GB per sample). The paired-end reads were 150 base pairs in length. Primary sequence data were generated by applying the Illumina DRAGEN BCL Convert 07.021.645.4.0.3 pipeline.

#### 2.6.3 Sequence read quality control and mapping

The initial bioinformatic analysis involved demultiplexing and quality control. Subsequently, the data were processed using an RNAseq expression analysis workflow that included trimming, alignment, transcript assembly, feature quantification, and differential expression analysis. The sequence files were generated in the standard FASTQ format. All 20 samples showed a base sequence quality with >93% bases exceeding Q30 across all samples. The reads were screened for the presence of adapters, cross-species contamination, and overrepresented sequences. Subsequently, the high-quality sequence reads were aligned to the reference genome (*Gallus gallus* GRCg7b) using the STAR aligner (v2.5.3a), in line with the instructions provided in the manual (available at https://github.com/alexdobin/STAR) (13). The resulting alignment files were generated in compressed binary alignment map (BAM) formats. The sequencing data have been deposited in the NCBI Sequence Read Archive under BioProject accession PRJNA1417341. Read counts were summarised from uniquely mapped reads per annotated gene using featureCounts (v1.5.3) in R for downstream analysis with the edgeR package (14). The StringTie tool v2.1.4 was used to assemble the transcripts, applying the read alignment and reference annotation-based assembly (RABT) option (15). The RefSeq annotation, which contained both coding and non-coding annotations for the genome, served as a guide (16). On average, 78.2% of the paired reads were successfully mapped to the reference genome, with 65.1% uniquely annotated to single genes.

#### 2.6.4 Differentially expressed genes and functional analyses

The statistical analyses were performed according to the methodology outlined by Niknafs et al. (17). In short, treatments were compared pairwise for differential expression analysis in EdgeR (version 4.0.9) using the R package 4.3.1. EdgeR is a tool designed to identify and quantify differential expression in RNAseq data by utilising the counts of uniquely mapped reads for each gene of *G. gallus*. The default normalisation method in EdgeR was used to normalise sample counts. A generalised linear model (GLM) was used to quantify differential expression between treatment groups. Differentially expressed genes (DEGs) were determined based on a) a threshold of false discovery rate (FDR)<0.05 and −0.5 > log FC > 0.5, and b) a threshold of raw *p*-value<0.05 and −0.5 > log FC > 0.5 for comparing carvacrol-treated to saline-treated groups (T3 versus T1; T4 versus T2). Differential expression results were interpreted based on FDR-adjusted *p*-values to control multiple testing. In addition, unadjusted *p*-values were used in an exploratory manner to identify broader pathway-level trends. As this approach increases the risk of false-positive findings, results derived from unadjusted *p*-values should be interpreted with caution and considered hypothesis-generating rather than confirmatory. For pathway enrichment analyses, the DEGs between the treatment groups were used as input. Enrichment and functional analyses were conducted using the Database for Annotation, Visualization and Integrated Discovery (DAVID), applying a significance pathway enrichment threshold of *p*< 0.01 (18). Enriched metabolic pathways and terms from the Gene Ontology Biological Processes (GO BP) and Kyoto Encyclopedia of Genes and Genomes (KEGG) databases were integrated to improve the understanding of the functional implications of the DEGs identified between the pairwise comparisons of treatment groups (19, 20).

## 3 Results

### 3.1 Performance

The results of the *in ovo* delivery of carvacrol at performance at d0 and d7 have been presented in our previous work (21). Briefly, none of the measured parameters (hatchability, navel quality and chicken quality at hatch, BW, BWG, or FCR) were affected by the *in ovo* delivery of carvacrol (*p* > 0.05), except for FI at d7, which was 11 g lower after carvacrol injection compared to the control treatment (*p* = 0.02).

Chickens received a repeated challenge with LPS at d7 and d14. At d14, before the second challenge, no interaction effects or main effects of *in ovo* treatment and/or challenge (*p* > 0.05) were found on BW, BWG, FI, and FCR between d0 and d14 and between d7 and d14 (**Table 1**).

**Table 1:** Performance results (LSmeans ± pooled SEM) at d14, including body weight (BW), body weight gain (BWG), feed intake (FI), and feed conversion ratio (FCR) measured between d0 and d14 and between d7 and d14.

*In ovo*	Challenge	BW d7 (g)	BW d14 (g)	BWG d0–d14 (g)	BWG d7–d14 (g)	FI d0–d14 (g)	FI d7–d14 (g)	FCR d0–d14 (g/g)	FCR d7–d14 (g/g)
Saline		227	587	544	360	693	502	1.27	1.40
Carvacrol		221	570	528	352	677	494	1.28	1.40
SEM		2.47	7.48	7.54	5.22	7.74	6.13	0.01	0.01
	Saline		581	539	362	689	504	1.28	1.39
	LPS		576	533	350	677	493	1.27	1.41
	SEM		7.48	7.54	5.22	7.74	6.13	0.01	0.01
Saline	Saline		585	542	362	695	505	1.28	1.39
	LPS		589	546	358	691	500	1.27	1.40
Carvacrol	Saline		578	535	363	684	503	1.28	1.39
	LPS		563	520	342	664	485	1.28	1.42
SEM			10.57	10.67	7.38	10.94	8.68	0.01	0.01
*P*-value									
*In ovo*		0.10	0.14	0.15	0.31	0.10	0.34	0.85	0.62
Challenge			0.60	0.60	0.13	0.29	0.22	0.45	0.23
*In ovo* × Challenge			0.39	0.38	0.28	0.48	0.45	0.45	0.34

All measurements between d7 and d14 were taken before the lipopolysaccharide (LPS) challenge on both days. Chickens received an *in ovo* injection of saline or carvacrol into the amniotic fluid at embryonic d17.5, followed by post-hatch saline or LPS challenges at d7 and 14.

At 6 h after the second challenge (d14), no interactions between *in ovo* treatment and challenge were found on BW, BWG, and relative organ percentages (*p* > 0.05, **Table 2**). However, the LPS challenge resulted in a lower BW (Δ 32 g, *p*< 0.001), 6 h BWG (Δ 33 g, *p*< 0.001), and a higher percentage of spleen relative to BW (Δ 0.03%, *p*< 0.001).

**Table 2:** Body weight (BW), 6-h body weight gain (BWG + 6 h), and relative organ weights (liver, bursa of Fabricius, and spleen; % of BW) measured at d14 (LSmeans ± pooled SEM).

*In ovo*	Challenge	BW (g)	BWG + 6 h (g)	Liver (%)	Bursa (%)	Spleen (%)
Saline		592	15	3.56	0.18	0.08
Carvacrol		593	16	3.51	0.20	0.09
SEM		3.10	2.81	0.07	0.01	0.01
	Saline	608^a^	32^a^	3.47	0.20	0.07^b^
	LPS	576^b^	−1^b^	3.59	0.18	0.10^a^
	SEM	2.98	2.81	0.07	0.01	0.01
Saline	Saline	610	33	3.56	0.19	0.07
	LPS	573	−4	3.56	0.18	0.09
Carvacrol	Saline	607	30	3.39	0.22	0.07
	LPS	579	2	3.63	0.18	0.10
SEM		4.31	3.98	0.09	0.01	0.01
*P*-value						
*In ovo*		0.79	0.69	0.61	0.30	0.21
Challenge		<0.001	<0.001	0.20	0.12	<0.001
*In ovo* × Challenge		0.37	0.30	0.28	0.34	0.20

^a,b^ LSmeans within a column and factor lacking a common superscript differ (*p* ≤ 0.05).

All measurements were taken 6 h after the second lipopolysaccharide (LPS) challenge. Chickens received an *in ovo* injection of saline or carvacrol into the amniotic fluid at embryonic d17.5, followed by post-hatch saline or LPS challenges at d7 and d14.

### 3.2 Transcriptomic analysis

A total of 11,784 transcripts were identified from all splenic samples. The pairwise comparison of the gene expression data resulted in the identification of groups of DEGs (**Table 3**) when comparing CTRLPS–CTRCTR, CARLPS–CARCTR, or CARLPS–CTRLPS (FDR< 0.05, −0.5 > logFC > 0.5). For the comparison CARCTR vs. CTRCTR, no DEGs were found. The number of DEGs allowed for functional enrichment analysis for the comparisons between the challenged and non-challenged treatments that received either *in ovo* saline or carvacrol (“effects of LPS”; CTRLPS vs. CTRCTR or CARLPS vs. CARCTR). For the comparisons between the *in ovo* delivery of carvacrol and saline (“effects of carvacrol”; CARCTR vs. CTRCTR or CARLPS vs. CTRLPS), the number of DEGs (0 or 52, respectively) was insufficient. The comparison CARLPS vs. CTRCTR was considered to evaluate the added effect of both carvacrol and LPS challenge compared to the control treatment. For the comparison CARLPS vs. CTRCTR, a total of 1,906 DEGs were identified. However, 87.4% of these genes (687 out of 786) overlapped with those already identified in the comparison CTRLPS vs. CTRCTR. Because this comparison did not provide additional biological insight beyond the effect of the LPS challenge alone, it was not considered further. The final comparison option (CTRLPS vs. CARCTR) was not assessed because it was not related to the aim of this study.

**Table 3:** Differentially expressed genes (DEGs; FDR< 0.05, −0.5 > logFC > 0.5) in transcriptomic comparisons of splenic samples from 14-day-old chickens receiving either saline (CTR) or carvacrol (CAR) *in ovo* at embryonic d17.5 and a repeated saline (CTR) or lipopolysaccharide (LPS) challenge at d7 and d14 post-hatching.

Category	CTRLPS vs. CTRCTR	CARLPS vs. CARCTR	CARCTR vs. CTRCTR	CARLPS vs. CTRLPS
Total DEGs	786	1,832	0	52
Increased	424	880	0	20
Decreased	362	952	0	32
Not DEGs	10,998	9,952	11,784	11,732

This resulted in four treatment groups: CTRCTR, CTRLPS, CARCTR, and CARLPS.

FDR, false discovery rate.

For the “effects of LPS” comparisons (CTRLPS vs. CTRCTR and CARLPS vs. CARCTR), GO and KEGG analyses showed that the DEGs significantly enriched (*p*< 0.01) pathways related to immunomodulation (**Tables 4**, **5**), with most DEGs being upregulated by the LPS challenge.

**Table 4:** Immunomodulatory pathways (*p*< 0.01) enriched in the spleen of 14-day-old broiler chickens in the comparison between CTRLPS and CTRCTR.

Enrichment					DEG enriching the pathways	
Database	Immune pathway terms	*p*-Value	% enriched	Fold enrichment	Upregulated by LPS	Downregulated by LPS
GO	Immune response	0.000	3.39	3.45	17	9
	Inflammatory response	0.000	2.74	3.48	17	4
	Cellular response to lipopolysaccharide	0.000	1.30	4.29	9	1
	Chemokine-mediated signalling pathway	0.002	0.78	6.25	4	2
	Neutrophil chemotaxis	0.002	0.91	4.86	6	1
	Chemotaxis	0.002	1.04	4.17	4	4
	Antimicrobial humoral immune response mediated by antimicrobial peptide	0.005	0.65	6.63	5	0
	Positive regulation of T-cell activation	0.007	0.65	6.08	2	3
	Peptide antigen assembly with MHC class II protein complex	0.009	0.52	8.34	1	3
	Positive regulation of immune response	0.009	0.52	8.34	2	2
KEGG	Cytokine–cytokine receptor interaction	0.000	4.17	3.03	23	9
	Phagosome	0.000	2.61	2.63	15	5

Chickens received an *in ovo* injection of saline (CTR) at embryonic d17.5 and post-hatch saline (CTR) or lipopolysaccharide (LPS) challenges at d7 and d14, resulting in the treatment groups CTRCTR and CTRLPS. Input differentially expressed genes (DEGs) were identified as false discovery rate (FDR)<0.05 and −0.5 > logFC > 0.5. Databases used for pathway enrichment were Gene Ontology (GO) and Kyoto Encyclopedia of Genes and Genomes (KEGG). A full list of genes is provided in **Supplementary Table S2**, and all enriched physiological pathways (*p*< 0.01) are listed in **Supplementary Table S1**.

**Table 5:** Immunomodulatory pathways (*p*< 0.01) enriched in the spleen of 14-day-old broiler chickens in the comparison between CARLPS and CARCTR.

Enrichment					DEG enriching the pathways	
Database	Immune pathway terms	*p*-Value	% enriched	Fold enrichment	Upregulated by LPS	Downregulated by LPS
GO	Immune response	0.000	3.21	3.27	42	15
	Inflammatory response	0.000	2.25	2.86	32	8
	Neutrophil chemotaxis	0.000	0.84	4.50	14	1
	Chemokine-mediated signalling pathway	0.000	0.68	5.40	10	2
	Cellular response to lipopolysaccharide	0.000	0.96	3.15	15	2
	Innate immune response	0.000	1.69	2.15	24	6
	Response to lipopolysaccharide	0.000	0.68	3.44	11	1
	Lymphocyte chemotaxis	0.001	0.39	4.90	6	1
	Cytokine-mediated signalling pathway	0.002	0.90	2.34	12	4
	Monocyte chemotaxis	0.002	0.45	3.88	7	1
	Positive regulation of tumour necrosis factor production	0.002	0.45	3.88	7	1
	Defence response to virus	0.002	0.68	2.70	12	0
	Chemotaxis	0.002	0.68	2.70	8	4
	Cellular response to interleukin-1	0.006	0.39	3.68	6	1
	Positive regulation of interleukin-6 production	0.006	0.39	3.68	6	1
	Acute-phase response	0.007	0.28	5.25	5	0
	Negative chemotaxis	0.008	0.51	2.84	3	6
	Negative regulation of the apoptotic process	0.009	1.52	1.65	20	7
KEGG	Cytokine–cytokine receptor interaction	0.000	3.94	3.03	55	15
	Influenza A	0.003	1.63	1.74	24	5
	NOD-like receptor signalling pathway	0.005	1.69	1.66	28	2
	p53 signalling pathway	0.009	1.07	1.86	10	9

Chickens received an *in ovo* injection of carvacrol (CAR) at embryonic d17.5 and post-hatch saline (CTR) or lipopolysaccharide (LPS) challenges at d7 and d14, resulting in the treatment groups CARCTR and CARLPS. Input differentially expressed genes (DEGs) were identified as false discovery rate (FDR)<0.05 and −0.5 > logFC > 0.5. Databases used for pathway enrichment were Gene Ontology (GO) and Kyoto Encyclopedia of Genes and Genomes (KEGG). A full list of genes is provided in **Supplementary Table S4**, and all enriched physiological pathways (*p*< 0.01) are listed in **Supplementary Table S3**.

A less stringent cut-off was used based on unadjusted (raw) *p*-values (*p*< 0.05 and −0.5 > logFC > 0.5) in the assessment of the “effects of carvacrol” (CARCTR vs. CTRCTR and CARLPS vs. CTRLPS) to provide sufficient input for functional enrichment analysis (**Table 6**).

**Table 6:** Numbers of differentially expressed genes (DEGs; *p*-value<0.05, −0.5 > logFC > 0.5) in transcriptomic comparisons of splenic samples from 14-day-old chickens receiving either saline (CTR) or carvacrol (CAR) *in ovo* at embryonic d17.5 and a repeated saline (CTR) or lipopolysaccharide (LPS) challenge at d7 and d14 post-hatching.

Category	CARCTR vs CTRCTR	CARLPS vs CTRLPS
Total DEGs	89	720
Increased	50	336
Decreased	39	384
Not DEGs	11,695	11,064

This resulted in four treatment groups: CTRCTR, CTRLPS, CARCTR, and CARLPS.

For the effects of carvacrol compared to saline after an LPS challenge (CARLPS vs. CTRLPS), GO and KEGG analyses showed that the DEGs significantly enriched (*p*< 0.01) pathways related to immunomodulation (**Table 7**), with most DEGs being upregulated by the *in ovo* delivery of carvacrol. For the effects of carvacrol compared to saline in the absence of LPS (saline-challenged), no immune-related pathways were found to be enriched (*p* > 0.01).

**Table 7:** Immunomodulatory pathways (*p*< 0.01) enriched in the spleen of 14-day-old broiler chickens in the comparison between CARLPS and CTRLPS.

Enrichment						DEG enriching the pathways	
Database	Immune pathway terms	*p*-Value	% enriched	Fold enrichment	DEGs count	Upregulated by carvacrol	Downregulated by carvacrol
GO	Defence response to virus	0.000	1.85	7.69	13	CD40, DHX58, IFIH1, IFIT5, IL6, IRF7, MLKL, MX1, OASL, RSAD2, SAMHD1, TMEM173	AICDA
	Immune response	0.000	3.13	3.31	22	AGTR1, CCL19, CCL20, CCL4, CCL5, CD40, CSF3, CTSV, CX3CL1, CX3CR1, DMB1, IL6, PGR2/3, TGFBR3, TLR15, TLR3, TNFSF10	APLNR, CD86, IL7, LIF, TNFSF11
	Cellular response to interleukin-1	0.000	0.99	9.66	7	CCL4, CCL5, CCL19, CCL20, CD40, CX3CL1, RORA	None
	Positive regulation of ERK1 and ERK2 cascade	0.000	1.70	3.68	12	CCL4, CCL5, CCL19, CCL20, CCN2, CD4, CX3CL1, PDGFA, THPO	BMPER, HTR2C, PDGFC
	Cellular response to tumour necrosis factor	0.001	0.99	5.52	7	CCL4, CCL5, CCL19, CCL20, CD40, CX3CL1, RORA	None
	Lymphocyte chemotaxis	0.001	0.71	9.20	5	CCL4, CCL5, CCL19, CCL20, CX3CL1	None
	Cellular response to interferon-gamma	0.004	0.85	5.23	6	CCL4, CCL5, CCL19, CCL20, CX3CL1, NOS2	None
	Monocyte chemotaxis	0.006	0.71	6.37	5	CCL4, CCL5, CCL19, CCL20, CX3CL1	None
	Chemokine-mediated signalling pathway	0.008	0.71	5.92	5	CCL4, CCL5, CCL19, CCL20, CX3CL1	None
KEGG	Cytokine–cytokine receptor interaction	0.000	3.84	3.22	27	CCL4, CCL5, CCL19, CCL20, CD4, CD40, CSF3, CX3CL1, CX3CR1, EDA2R, GHR, IL6, IL12RB2, IL13RA2, IL20RA, THPO, TNFRSF10B, TNFRSF11B, TNFRSF25, TNFRSF6B, TNFRSF9, TNFSF10	CNTFR, IL7, LIF, NGFR, TNFSF11
	Cytosolic DNA-sensing pathway	0.010	1.14	3.24	8	CCL4, CCL5, IL6, IRF7, MLKL, SAMHD1, TMEM173	DNASE2B

AGTR, angiotensin II receptor type 1; AICDA, activation-induced cytidine deaminase; APLNR, apelin receptor; BMPER, BMP-binding endothelial regulator; CCL19, C–C motif chemokine ligand 19; CCL20, C–C motif chemokine ligand 20; CCL4, C–C motif chemokine ligand 4; CCL5, C–C motif chemokine ligand 5; CCN2, cellular communication network factor 2; CD4, CD4 molecule; CD40, CD40 molecule; CD86, CD86 molecule; CNTFR, ciliary neurotrophic factor receptor; CSF3, colony-stimulating factor 3; CTSV, cathepsin V; CX3CL1, C-X3-C motif chemokine ligand 1; DHX58, DExH-box helicase 58; DMB1, major histocompatibility complex B, class II beta chain; DNASE2B, deoxyribonuclease 2 beta; EDA2R, ectodysplasin A2 receptor; GHR, growth hormone receptor; HTR2C, 5-hydroxytryptamine receptor 2C; IFIH1, interferon-induced with helicase C domain 1; IFIT5, interferon-induced protein with tetratricopeptide repeats 5; IL12RB2, interleukin 12 receptor subunit beta 2; IL13RA2, interleukin 13 receptor subunit alpha 2; IL20RA, interleukin 20 receptor subunit alpha; IL6, interleukin 6; IL7, interleukin 7; IRF7, interferon regulatory factor 7; LIF, leukaemia inhibitory factor; MLKL, mixed lineage kinase domain-like pseudokinase; MX1, myxovirus resistance 1; NGFR, nerve growth factor receptor; NOS2, nitric oxide synthase 2; OASL, 2′-5′-oligoadenylate synthetase-like; PDGFA, platelet-derived growth factor subunit A; PDGFC, platelet-derived growth factor C; PGR2/3, tandem PRG2/PRG3 gene pair; RORA, RAR-related orphan receptor A; RSAD2, radical *S*-adenosyl methionine domain-containing 2; SAMHD1, SAM and HD domain-containing deoxynucleoside triphosphate triphosphohydrolase 1; TGFBR3, transforming growth factor beta receptor 3; THPO, thrombopoietin; TLR15, toll-like receptor 15; TLR3, toll-like receptor 3; TMEM173, transmembrane protein 173; TNFRSF10B, tumour necrosis factor receptor superfamily member 10B; TNFRSF11B, tumour necrosis factor receptor superfamily member 11B; TNFRSF25, tumour necrosis factor receptor superfamily member 25; TNFRSF6B, tumour necrosis factor receptor superfamily member 6B; TNFRSF9, tumour necrosis factor receptor superfamily member 9; TNFSF10, tumour necrosis factor superfamily member 10; TNFSF11, tumour necrosis factor superfamily member 11.

Chickens received an in ovo injection of carvacrol (CAR) or saline (CTR) into the amniotic fluid at embryonic d17.5, and both groups received lipopolysaccharide (LPS) challenges at d7 and d14 post-hatching, resulting in the treatment groups CARLPS and CTRLPS. Input differentially expressed genes (DEGs) were identified as *p*-value<0.05 and −0.5 > logFC > 0.5. Databases used for pathway enrichment were Gene Ontology (GO) and Kyoto Encyclopedia of Genes and Genomes (KEGG). All enriched physiological pathways (*p*< 0.01) are listed in **Supplementary Table S5**.

## 4 Discussion

**Figure 2:**
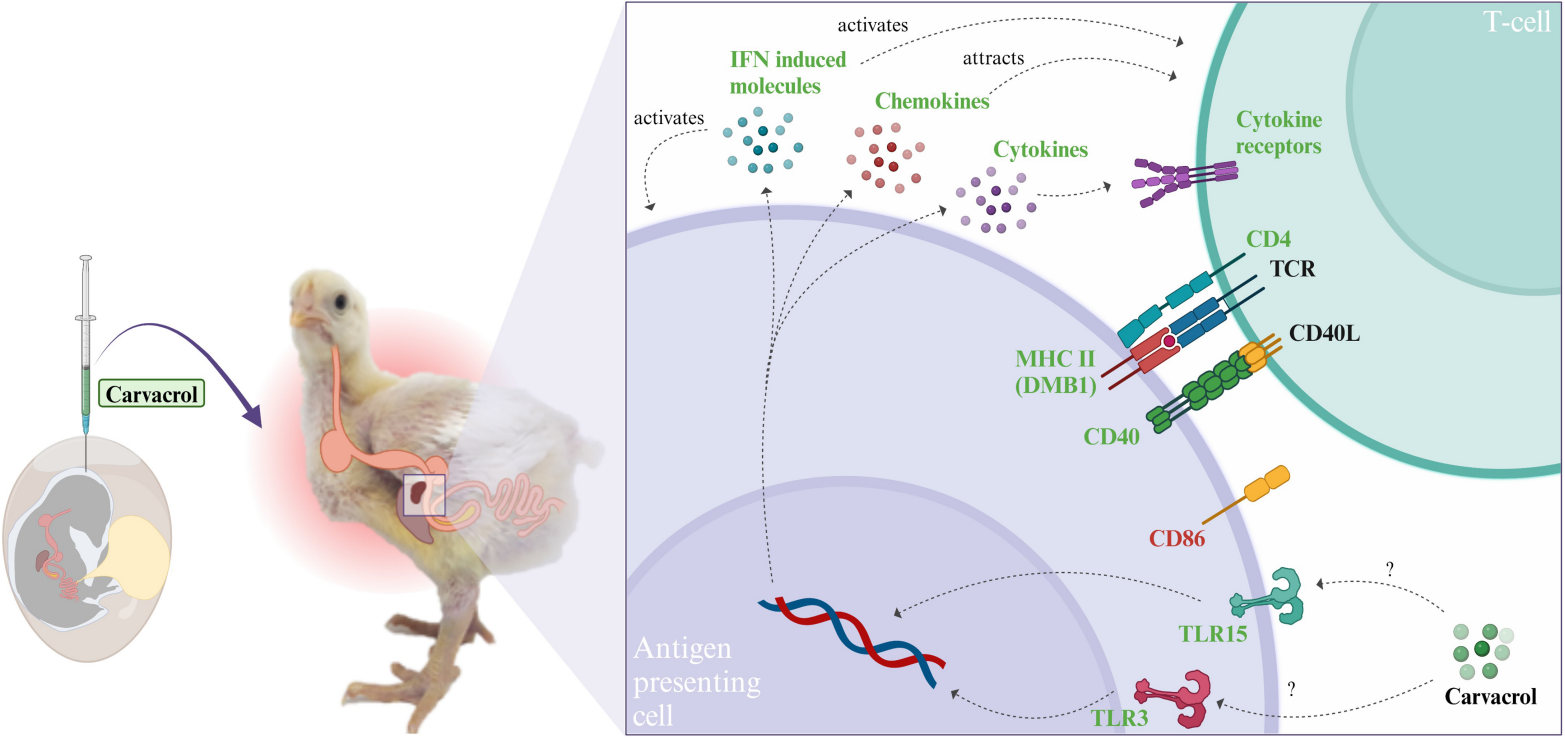
Integration of genes enriching metabolic pathways to illustrate functional implications of differentially expressed genes (DEGs) in the spleen of broiler chickens at d14 post-hatch. Chickens received carvacrol *in ovo* at embryonic d17.5 and were challenged with lipopolysaccharide (LPS) at d7 and d14. The figure shows differences between chickens receiving carvacrol *in ovo* compared to saline after the LPS challenge at d14. Metabolic pathways were identified across the Kyoto Encyclopedia of Genes and Genomes (KEGG) and Gene Ontology (GO) databases. Green indicates genes upregulated by carvacrol; red indicates genes downregulated by carvacrol. Created with BioRender.com. IFN, interferon; CD4, CD4 molecule; CD40, CD40 molecule; CD40L, CD40 ligand; CD86, CD86 molecule; TCR, T-cell receptor; TLR3, toll-like receptor 3; TLR15, toll-like receptor 15.

The current study aimed to assess the effects of the *in ovo* delivery of carvacrol on immune responses in 14-day-old broiler chickens following repeated LPS challenges on d7 and 14. LPS administration resulted in a lower BW and BWG at 6 h after the challenge on d14, accompanied by a higher relative spleen weight, indicating a negative impact from the LPS (22). The *in ovo* delivery of carvacrol did not mitigate the negative impact of LPS on BW or spleen weight. Given the observed impact of LPS on spleen weight, a comparative spleen transcriptomic analysis was performed to identify DEGs. Subsequent pathway enrichment analyses were conducted to investigate immune mechanisms. **Figure 2** shows our interpretation, and the integration of DEGs and pathways is shown in **Table 7**.

Initially, using a stringent *p*-value cut-off (FDR), the effects of the LPS challenge without or with carvacrol (CTRLPS vs. CTRCTR or CARLPS vs. CARCTR, respectively) were analysed, identifying enriched pathways associated with inflammatory mechanisms, suggesting that the LPS challenge had been successful in activating an immune response. Subsequently, a less stringent *p*-value cut-off (unadjusted *p-*value) was applied to compare the effects of carvacrol during non-challenged conditions (CARCTR vs. CTRCTR) or after the LPS challenge (CARLPS vs. CTRLPS). Only the second comparison resulted in the identification of sufficient DEGs for pathway enrichment analysis of immune-related pathways. This suggests that the *in ovo* delivery of carvacrol may modulate immune responses specifically toward LPS challenge while showing no impact under non-challenged conditions at d14 post-hatch. A potential overstimulation of the immune system could adversely affect performance, yet in broiler chickens selected for growth with compromised immune responsiveness, modulated immune responses could prove advantageous (3, 4, 23). Moreover, the limited differential gene expression in the absence of LPS challenge indicates that carvacrol likely does not induce immune responses under neutral conditions, suggesting that its role in modulating immune mechanisms is more pronounced during immune challenges when an enhanced response could be beneficial. This is in contrast with our previous work, where the *in ovo* delivery of carvacrol did influence immune-related gene expression, particularly around the time of hatch (11, 21, 24). However, those effects were observed at an earlier developmental stage and in different tissues than the current study. Together, this suggests that the impact of carvacrol on immune gene regulation may be age- and context-dependent, with more pronounced effects during early immune development or when an immune challenge is present.

Specifically, cytokine and chemokine signalling appeared to be influenced by the *in ovo* delivery of carvacrol during the LPS challenge, as shown by enrichment of pathways such as “*Cellular response to interleukin-1*”, “*Cellular response to tumour necrosis factor*”, “*Cellular response to interferon-gamma*”, “*Cytokine–cytokine receptor interaction*”, “*Lymphocyte chemotaxis*”, “*Monocyte chemotaxis*”, and “*Chemokine-mediated signalling pathway*”. Similar pathway activation has been observed during acute innate responses in chickens exposed to LPS (25, 26). However, the observed enrichment in the current study, where the *in ovo* delivery of carvacrol was combined with the LPS challenge, indicated that the essential oil compound modified the LPS-induced response rather than initiating it.

When looking at the DEGs enriching these pathways, the majority encoded cytokines, chemokines, and their receptors. Most of these immune-related DEGs could be grouped into chemokines (CCL4, CCL5, CCL19, CCL20, and CX3CL1), interferon-induced genes (IFIH1, IFIT5, IRF7, MX1, RSAD2, and TMEM173), interleukins and their receptors (IL12RB2, IL13RA2, IL20RA, IL6, IL7, and LIF), tumour necrosis factor family members and their receptors (TNFSF10, TNFSF11, TNFRSF10B, TNFRSF11B, TNFRSF25, TNFRSF6B, and TNFRSF9), transforming growth factor beta receptor (TGFBR3), toll-like receptors (TLR3 and TLR15), clusters of differentiation (CD4, CD40, and CD86), and major histocompatibility complex class II molecule DMB1. These patterns are again consistent with typical LPS-induced immune activation in the spleen, indicating that carvacrol may amplify baseline LPS responses (27, 28). Except for IL7, LIF, TNFSF11, and CD86, these immune-related genes were upregulated by the *in ovo* delivery of carvacrol during the LPS challenge, indicating the potential of the *in ovo* delivery of carvacrol to modulate immune activation during a post-hatching challenge. This aligns with our earlier findings where the *in ovo* delivery of carvacrol influenced immune-related gene expression around hatching, but differs in age and target tissue, suggesting that the immunomodulatory effects of essential oil compounds are dependent on developmental stage, tissue, and the presence of an immune challenge (11, 24).

Based on the observed DEGs and enriched pathways, the *in ovo* delivery of carvacrol may have modulated immune responses through multiple mechanisms. After *in ovo* delivery in the amniotic fluid at E17.5, carvacrol is known to migrate and accumulate in the yolk (29). However, excessive amounts of carvacrol *in ovo* may be toxic for the developing embryo (30). Carvacrol is known to impact toll-like receptors (TLRs) (7, 31, 32), and the upregulation of TLR3 (responsive to viral antigens) and TLR15 (unique for avian species and responsive to bacterial antigens) (33) could explain the initial mode of action. The activation of these TLRs may, in turn, have triggered transcription factors, leading to nuclear transcription of chemokines and cytokine-related molecules (34). Specifically, TLR3 is known to activate innate immune responses through downstream signalling pathways, leading to an increase in antiviral responses through interferon-related molecules (35), of which several were found to be upregulated simultaneously in the current study.

The increased expression of chemokine and cytokine mediators (IL, TNF, and TGFβ) suggests enhanced migration, activation, and maturation of antigen-presenting cells (APC) and T cells in response to LPS challenge (36, 37). Furthermore, the upregulation of both co-stimulatory molecule CD40 and DMB1 (a component of splenic major histocompatibility complex II), which are both found on APC, suggests enhanced antigen presentation to T cells. This is particularly interesting since CD4, part of the T-cell receptor, was also found to be upregulated. Subsequently, this could result in enhanced T-cell activation and differentiation (38–40). The downregulation of several genes associated with T-cell activation (IL7, LIF, TNFSF11, and CD86) suggests that while immune responses are activated, regulatory mechanisms may also be at play. Overall, the *in ovo* delivery of carvacrol appears to promote T-cell activation and differentiation through APC activation.

Oregano essential oil and its main functional compound carvacrol are known to have immunomodulatory effects when supplemented to broiler chickens’ diets post-hatching (6, 31, 41–44). Interestingly, carvacrol has previously been recognised for its anti-inflammatory properties, including the suppression of TLR4, NF-κB, and pro-inflammatory cytokines (7, 45). However, our current findings suggest otherwise—the *in ovo* delivery of carvacrol appears to stimulate immune responses rather than suppress them, which could potentially be attributed to the stage of supplementation (pre- versus post-hatch). In our previous work, *in ovo* carvacrol was associated with anti-inflammatory effects at d7, whereas this effect was not apparent at d14, suggesting that the immunomodulatory effects may be time-dependent during early development (21). In addition, anti-inflammatory effects have been reported following post-hatch carvacrol supplementation, further indicating that immune modulation depends on timing, route of administration, and biological context (7). This aligns with our earlier research on the *in ovo* delivery of carvacrol, where we have shown that after delivery at E17.5, carvacrol migrates to the yolk and has the potential to enhance antimicrobial defence mechanisms in the yolk sac (24, 29). Additionally, our observations indicated that the *in ovo* delivery of carvacrol without subsequent immune challenge showed immune-activating effects in the jejunum and yolk sac before hatching (E19.5), suggesting that the *in ovo* route may elicit a stimulating rather than inhibitory response (11). In contrast, the carvacrol treatment in combination with an LPS challenge at d7 mitigated the upregulation of splenic inflammatory genes (TLR4, NF-κB, IL-1, and TNF-α), indicating some immunosuppressive effects (21) at this earlier post-hatch stage. The apparent differences between these responses and the current findings at d14 may be explained by the limited maturation of the broiler immune system during the first 2 weeks post-hatching (5), highlighting the time- and context-dependent nature of carvacrol’s immunomodulatory effects.

Several limitations of the present study should be considered when interpreting the results. While the observed changes represent increased transcriptional responsiveness to LPS, they do not necessarily indicate improved immune function or performance. Furthermore, this study relies on transcriptomic analysis to assess immune responses. Immune outcomes at metabolic or protein levels, such as plasma cytokine levels, functional immune cell activation, or histological changes, were not evaluated. As a result, the mechanistic interpretation of the outcomes should be limited to gene expression, and the findings should be considered mainly as descriptive and hypothesis-generating. LPS was used as a standardised stimulus to induce systemic immune activation, with challenges at d7 and d14 included to assess immune responses during the first 2 weeks post-hatch and to represent repeated inflammatory stimulation rather than a single acute event. Repeated immune challenges are likely to occur under commercial conditions. In addition, the *in ovo* carvacrol dose was selected based on previous dose–response work with oregano essential oil (~75% carvacrol), which identified an embryonic safety margin, although potential long-term safety effects beyond the experimental period were not assessed (30).

Immune responses in the present study were assessed exclusively at the transcriptomic level in splenic tissue and at a single time point (6 h after the second LPS challenge). Consequently, the observed changes did not capture the temporal dynamics of the immune response. Although pathways related to T-cell function were enriched, this reflects transcriptional signatures rather than functional T-cell activation. Despite immune-related transcriptional changes, no improvements in growth performance were observed, indicating that enriched immune gene expression does not necessarily translate into short-term production benefits. In addition, performance-focused studies would usually require a higher number of replications, which were not available in the experimental design used. No adverse welfare indicators such as impaired hatchability, poor chick quality, or reduced survival were observed following *in ovo* carvacrol administration; however, welfare outcomes were not directly assessed and therefore remain an important area for future investigation. Furthermore, the study was conducted using broiler chickens with Ross 308 genetics and under controlled experimental conditions. Thus, potential breed effects or other environmental influences were outside the scope of this research project. Carvacrol bioavailability and metabolism following *in ovo* delivery were not assessed but should be addressed in future research. Together, these factors place the findings in an exploratory, hypothesis-generating context. Overall, the findings of the current study suggest that the *in ovo* delivery of carvacrol may modulate immune pathways persisting until at least 14 days post-hatching, potentially enhancing defence mechanisms and regulating immune cell activity in response to LPS. This indicates that carvacrol may contribute to an increased state of immune preparedness, in which the immune system remains relatively inactive under neutral conditions but responds more rapidly and robustly when challenged.

In conclusion, the LPS challenge negatively impacted broiler chicken performance, but the *in ovo* delivery of carvacrol at E17.5 did not mitigate these effects 6 h after the second LPS challenge at d14 post-hatching. However, the *in ovo* delivery of carvacrol enriched immune pathways 6 h after the LPS challenge at d14 post-hatch, specifically by upregulating gene expression of cytokines, chemokines, and their receptors.

This study demonstrates that *in ovo* carvacrol can influence immune-related transcriptional responses to a later inflammatory challenge. The immunomodulatory effects of essential oils applied *in ovo* in broilers have been recently reviewed, showing increased bursal activity and serum levels of several immunoglobulins (46). Furthermore, the *in ovo* delivery of carvacrol on immune development and immune activation in the jejunum, bursa of Fabricius, and yolk sac was previously explored (11, 24). Overall, the current knowledge, including the results presented in this publication, offers a strategy to enhance early post-hatch disease resilience in broiler production. However, the effects presented were observed at the transcriptomic level and were not associated with performance changes. While early-life exposure to carvacrol altered immune-related gene expression following LPS challenge, further functional and longitudinal studies are required to determine health outcomes and applied performance trials.

## Data Availability

The original contributions presented in the study are publicly available. This data can be found here: https://www.ncbi.nlm.nih.gov/bioproject/PRJNA1417341.
