# Supplementary material for: In ovo carvacrol enriched inflammatory and T-cell transcriptional responses to Escherichia coli LPS in broiler chickens
Source: Front Immunol. 2026 Mar 3;17:1761404. doi: 10.3389/fimmu.2026.1761404 (PMC12992001; doi:10.3389/fimmu.2026.1761404)
Supplement: Supplementary file 1 [file DataSheet1.docx]

Supplementary Material

**Supplementary Table 1**. CTRLPS vs CTRCTR. All physiological pathways (*P* < 0.01) enriched in the spleen of broiler embryos at d14 post-hatching after in ovo delivery of saline at E17.5 and receiving lipopolysaccharide (LPS) at d7 and d14 post hatching. Input differentially expressed genes (DEG) were identified as FDR <0.05 and -0.5> logFC > 0.5. Databases used are Gene Ontology (GO) and Kyoto Encyclopedia of Genes and Genomes (KEGG).

| Database | Term | *P*-value | % enriched | Fold enrichment | Number of DEG |
| --- | --- | --- | --- | --- | --- |
| GO | immune response | 0.000 | 3.39 | 3.45 | 26 |
|  | mitotic sister chromatid segregation | 0.000 | 1.30 | 9.12 | 10 |
|  | cell division | 0.000 | 3.39 | 3.21 | 26 |
|  | inflammatory response | 0.000 | 2.74 | 3.48 | 21 |
|  | G-protein coupled receptor signalling pathway | 0.000 | 3.78 | 2.66 | 29 |
|  | chromosome segregation | 0.000 | 1.69 | 4.99 | 13 |
|  | extracellular matrix organization | 0.000 | 2.09 | 3.59 | 16 |
|  | CENP-A containing nucleosome assembly | 0.000 | 0.78 | 9.73 | 6 |
|  | kinetochore assembly | 0.000 | 0.78 | 9.73 | 6 |
|  | mitotic cytokinesis | 0.000 | 1.17 | 4.86 | 9 |
|  | cellular response to lipopolysaccharide | 0.000 | 1.30 | 4.29 | 10 |
|  | microtubule-based movement | 0.001 | 1.56 | 3.37 | 12 |
|  | mitotic spindle assembly checkpoint | 0.001 | 0.91 | 6.01 | 7 |
|  | negative regulation of endopeptidase activity | 0.001 | 0.78 | 6.73 | 6 |
|  | mitotic chromosome condensation | 0.001 | 0.65 | 9.12 | 5 |
|  | chemokine-mediated signalling pathway | 0.002 | 0.78 | 6.25 | 6 |
|  | mitotic cell cycle | 0.002 | 1.43 | 3.21 | 11 |
|  | mitotic spindle organization | 0.002 | 1.04 | 4.32 | 8 |
|  | neutrophil chemotaxis | 0.002 | 0.91 | 4.86 | 7 |
|  | chemotaxis | 0.002 | 1.04 | 4.17 | 8 |
|  | high-density lipoprotein particle remodelling | 0.003 | 0.52 | 11.67 | 4 |
|  | metaphase plate congression | 0.003 | 0.52 | 11.67 | 4 |
|  | antimicrobial humoral immune response mediated by antimicrobial peptide | 0.005 | 0.65 | 6.63 | 5 |
|  | collagen catabolic process | 0.007 | 0.65 | 6.08 | 5 |
|  | positive regulation of T cell activation | 0.007 | 0.65 | 6.08 | 5 |
|  | peptide antigen assembly with MHC class II protein complex | 0.009 | 0.52 | 8.34 | 4 |
|  | positive regulation of immune response | 0.009 | 0.52 | 8.34 | 4 |
|  | G2/M transition of mitotic cell cycle | 0.010 | 0.78 | 4.38 | 6 |
|  |  |  |  |  |  |
| KEGG | Cytokine-cytokine receptor interaction | 0.000 | 4.17 | 3.03 | 32 |
|  | Neuroactive ligand-receptor interaction | 0.000 | 3.91 | 2.20 | 30 |
|  | Phagosome | 0.000 | 2.61 | 2.63 | 20 |
|  | Cell adhesion molecules | 0.001 | 2.09 | 2.52 | 16 |
|  | ECM-receptor interaction | 0.003 | 1.69 | 2.66 | 13 |

**Supplementary Table 2.** CTRLPS vs CTRCTR. Showing all genes involved in immunomodulatory pathways (*P* < 0.01) enriched in the spleen of broiler embryos at d14 post-hatching after in ovo delivery of saline at E17.5 and receiving lipopolysaccharide (LPS) at d7 and d14 post hatching. Input differentially expressed genes (DEG) were identified as FDR <0.05 and -0.5> logFC > 0.5. Databases used are Gene Ontology (GO) and Kyoto Encyclopedia of Genes and Genomes (KEGG).

|  |  |  |  |  |  | DEG enriching the pathways | |
| --- | --- | --- | --- | --- | --- | --- | --- |
| Database | Pathway Terms | *P*-value | % enriched | Fold enrichment | Number of DEG | Upregulated by LPS | Downregulated by LPS |
| GO | Immune response | 0.000 | 3.39 | 3.45 | 26 | ACKR2, ACKR4, B2M, BDKRB1, BF1, CTLA4, CXCL13L2, CXCL13L3, FAS, FTH1, IL10, IL1B, IL8L1, TLR1B, TLR2A, TNFSF8, CD274 | BLB1, CCL17, CCR4, CCR6, CCR8L, CXCL12, DMA, DMB2, XCR1 |
|  | Inflammatory response | 0.000 | 2.74 | 3.48 | 21 | ADAM8, BDKRB1, CD44, CXCL13, CXCL13L2, CXCL13L3, EXFABP, IL1B, IL1R2, IL1RAP, IL2RA, IL8L1, PTAFR, TLR1B, TLR2A, TNFRSF4, PTGFR | CCL17, CCR4, KIT, RARRES2 |
|  | Cellular response to lipopolysaccharide | 0.000 | 1.30 | 4.29 | 10 | CD274, CXCL13, CXCL13L2, CXCL13L3, IL1B, IL8L1, TNIP3, ZC3H12A, IL10 | HMGB2 |
|  | Chemokine-mediated signalling pathway | 0.002 | 0.78 | 6.25 | 6 | CXCL13, CXCL13L2, CXCL13L3, IL8L1 | CCL17, CXCL12 |
|  | Neutrophil chemotaxis | 0.002 | 0.91 | 4.86 | 7 | BDKRB1, CXCL13, CXCL13L2, CXCL13L3, IL8L1, LGALS3 | CCL17 |
|  | Chemotaxis | 0.002 | 1.04 | 4.17 | 8 | ACKR2, ACKR4, PROK2, PTAFR | CCR4, CCR6, CCR8L, XCR1 |
|  | Antimicrobial humoral immune response mediated by antimicrobial peptide | 0.005 | 0.65 | 6.63 | 5 | CXCL13, CXCL13L2, CXCL13L3, GAPDH, IL8L1 |  |
|  | Positive regulation of T cell activation | 0.007 | 0.65 | 6.08 | 5 | B2M, THY1 | BLB1, DMA, DMB2 |
|  | Peptide antigen assembly with MHC class II protein complex | 0.009 | 0.52 | 8.34 | 4 | B2M | BLB1, DMA, DMB2 |
|  | Positive regulation of immune response | 0.009 | 0.52 | 8.34 | 4 | B2M, RSAD2 | BLB1, DMA |
|  |  |  |  |  |  |  |  |
| KEGG | Cytokine-cytokine receptor interaction | 0.000 | 4.17 | 3.03 | 32 | ACKR4, BMP7, CXCL13, CXCL13L2, CXCL13L3, FAS, IL10, IL10RA, IL13RA2, IL1B, IL1R2, IL1RAP, IL1RL1, IL21R, IL22, IL2RA, IL8L1, TNFRSF10B, TNFRSF11A, TNFRSF4, TNFRSF6B, TNFRSF8, TNFSF8 | CCL17, CCR4, CCR6, CCR8L, CXCL12, GDF11, GDF9, LEPR, XCR1 |
|  | Phagosome | 0.000 | 2.61 | 2.63 | 20 | ATP6V0C, ATP6V0D2, ATP6V1C2, ATP6V1G3, BF1, C1R, CALR, ITGB5, LAMP1, MMR1L4, PLA2R1, RAB7B, STX12, TCIRG1, TLR2A | BLB1, DMA, DMB2, MMR1L3, SCARB1 |

ACKR2 = atypical chemokine receptor 2; ACKR4 = atypical chemokine receptor 4; ADAM8 = ADAM metallopeptidase domain 8; ATP6V0C = ATPase H⁺ transporting V0 subunit c; ATP6V0D2 = ATPase H⁺ transporting V0 subunit d2; ATP6V1C2 = ATPase H⁺ transporting V1 subunit C2; ATP6V1G3 = ATPase H⁺ transporting V1 subunit G3; B2M = beta‑2‑microglobulin; BDKRB1 = bradykinin receptor B1; BF1 = major histocompatibility complex B, class I heavy chain BF1; BLB1 = major histocompatibility complex Y, class II beta BLB1; BMP7 = bone morphogenetic protein 7; C1R = complement C1r; CALR = calreticulin; CCL17 = C‑C motif chemokine ligand 17; CCR4 = C‑C motif chemokine receptor 4; CCR6 = C‑C motif chemokine receptor 6; CCR8L = C‑C chemokine receptor 8‑like; CD274 = CD274 molecule; CD44 = CD44 molecule; CTLA4 = cytotoxic T‑lymphocyte–associated protein 4; CXCL12 = C‑X‑C motif chemokine ligand 12; CXCL13 = C‑X‑C motif chemokine ligand 13; CXCL13L2 = C‑X‑C motif chemokine ligand 13‑like 2; CXCL13L3 = C‑X‑C motif chemokine ligand 13‑like 3; DMA = major histocompatibility complex B, class II alpha chain DMA; DMB2 = major histocompatibility complex B, class II beta chain DMB2; EXFABP = extracellular fatty acid‑binding protein; FAS = Fas cell surface death receptor; FTH1 = ferritin heavy chain 1; GAPDH = glyceraldehyde‑3‑phosphate dehydrogenase; GDF11 = growth differentiation factor 11; GDF9 = growth differentiation factor 9; HMGB2 = high mobility group box 2; IL10 = interleukin 10; IL10RA = interleukin 10 receptor subunit alpha; IL13RA2 = interleukin 13 receptor subunit alpha 2; IL1B = interleukin 1β; IL1R2 = interleukin 1 receptor type 2; IL1RAP = interleukin 1 receptor accessory protein; IL1RL1 = interleukin 1 receptor‑like 1; IL21R = interleukin 21 receptor; IL22 = interleukin 22; IL2RA = interleukin 2 receptor subunit alpha; IL8L1 = interleukin 8‑like 1; ITGB5 = integrin subunit beta 5; KIT = KIT proto‑oncogene, receptor tyrosine kinase; LAMP1 = lysosomal‑associated membrane protein 1; LEPR = leptin receptor; LGALS3 = galectin 3; MMR1L3 = macrophage mannose receptor 1‑like 3; MMR1L4 = macrophage mannose receptor 1‑like 4; PLA2R1 = phospholipase A2 receptor 1; PROK2 = prokineticin 2; PTAFR = platelet‑activating factor receptor; PTGFR = prostaglandin F receptor; RAB7B = RAB7B, member RAS oncogene family; RARRES2 = retinoic acid receptor responder 2; RSAD2 = radical S‑adenosyl methionine domain‑containing 2; SCARB1 = scavenger receptor class B member 1; STX12 = syntaxin 12; TCIRG1 = T‑cell immune regulator 1, ATPase H⁺ transporting V0 subunit a3; THY1 = Thy‑1 cell surface antigen; TLR1B = Toll‑like receptor 1 family member B; TLR2A = Toll‑like receptor 2A; TNFRSF10B = tumor necrosis factor receptor superfamily member 10B; TNFRSF11A = tumor necrosis factor receptor superfamily member 11A; TNFRSF4 = tumor necrosis factor receptor superfamily member 4; TNFRSF6B = tumor necrosis factor receptor superfamily member 6B; TNFRSF8 = tumor necrosis factor receptor superfamily member 8; TNFSF8 = tumor necrosis factor superfamily member 8; TNIP3 = TNFAIP3‑interacting protein 3; XCR1 = X‑C motif chemokine receptor 1; ZC3H12A = zinc finger CCCH‑type containing 12A.

**Supplementary Table 3.** CARLPS vs CARCTR. All physiological pathways (*P* < 0.01) enriched in the spleen of broiler embryos at d14 post-hatching after in ovo delivery of carvacrol at E17.5 and receiving lipopolysaccharide (LPS) at d7 and d14 post hatching. Input differentially expressed genes (DEG) were identified as FDR <0.05 and -0.5> logFC > 0.5. Databases used are Gene Ontology (GO) and Kyoto Encyclopedia of Genes and Genomes (KEGG).

| Database | Term | *P*-value | % enriched | Fold enrichment | Number of DEG |
| --- | --- | --- | --- | --- | --- |
| GO | immune response | 0.000 | 3.21 | 3.27 | 57 |
|  | inflammatory response | 0.000 | 2.25 | 2.86 | 40 |
|  | neutrophil chemotaxis | 0.000 | 0.84 | 4.50 | 15 |
|  | chemokine-mediated signalling pathway | 0.000 | 0.68 | 5.40 | 12 |
|  | extracellular matrix organization | 0.000 | 1.58 | 2.71 | 28 |
|  | G-protein coupled receptor signalling pathway | 0.000 | 2.70 | 1.90 | 48 |
|  | DNA replication | 0.000 | 1.52 | 2.47 | 27 |
|  | cellular response to lipopolysaccharide | 0.000 | 0.96 | 3.15 | 17 |
|  | innate immune response | 0.000 | 1.69 | 2.15 | 30 |
|  | DNA replication initiation | 0.000 | 0.62 | 3.85 | 11 |
|  | response to lipopolysaccharide | 0.000 | 0.68 | 3.44 | 12 |
|  | axon guidance | 0.000 | 1.58 | 2.05 | 28 |
|  | mitotic sister chromatid segregation | 0.000 | 0.56 | 3.94 | 10 |
|  | cell division | 0.000 | 1.97 | 1.87 | 35 |
|  | cell adhesion | 0.000 | 2.42 | 1.73 | 43 |
|  | double-strand break repair via break-induced replication | 0.000 | 0.39 | 5.51 | 7 |
|  | interstrand cross-link repair | 0.001 | 0.62 | 3.30 | 11 |
|  | kinetochore assembly | 0.001 | 0.39 | 4.90 | 7 |
|  | lymphocyte chemotaxis | 0.001 | 0.39 | 4.90 | 7 |
|  | DNA-dependent DNA replication | 0.001 | 0.51 | 3.78 | 9 |
|  | mitotic cell cycle | 0.001 | 1.01 | 2.27 | 18 |
|  | response to hypoxia | 0.001 | 0.84 | 2.49 | 15 |
|  | cytokine-mediated signalling pathway | 0.002 | 0.90 | 2.34 | 16 |
|  | monocyte chemotaxis | 0.002 | 0.45 | 3.88 | 8 |
|  | positive regulation of tumor necrosis factor production | 0.002 | 0.45 | 3.88 | 8 |
|  | homophilic cell adhesion via plasma membrane adhesion molecules | 0.002 | 1.18 | 2.00 | 21 |
|  | defense response to virus | 0.002 | 0.68 | 2.70 | 12 |
|  | chemotaxis | 0.002 | 0.68 | 2.70 | 12 |
|  | regulation of cell proliferation | 0.003 | 0.90 | 2.24 | 16 |
|  | DNA unwinding involved in DNA replication | 0.003 | 0.45 | 3.60 | 8 |
|  | positive regulation of cytosolic calcium ion concentration | 0.003 | 0.62 | 2.77 | 11 |
|  | bicarbonate transport | 0.004 | 0.34 | 4.73 | 6 |
|  | mitotic chromosome condensation | 0.004 | 0.34 | 4.73 | 6 |
|  | calcium-mediated signalling | 0.004 | 0.56 | 2.86 | 10 |
|  | modulation of synaptic transmission | 0.005 | 0.62 | 2.67 | 11 |
|  | protein refolding | 0.005 | 0.45 | 3.36 | 8 |
|  | cellular response to interleukin-1 | 0.006 | 0.39 | 3.68 | 7 |
|  | nucleobase-containing compound metabolic process | 0.006 | 0.39 | 3.68 | 7 |
|  | positive regulation of interleukin-6 production | 0.006 | 0.39 | 3.68 | 7 |
|  | CENP-A containing nucleosome assembly | 0.007 | 0.34 | 4.20 | 6 |
|  | response to organic substance | 0.007 | 0.34 | 4.20 | 6 |
|  | hydrogen peroxide catabolic process | 0.007 | 0.34 | 4.20 | 6 |
|  | acute-phase response | 0.007 | 0.28 | 5.25 | 5 |
|  | DNA repair | 0.008 | 1.63 | 1.63 | 29 |
|  | negative chemotaxis | 0.008 | 0.51 | 2.84 | 9 |
|  | male gonad development | 0.008 | 0.56 | 2.63 | 10 |
|  | negative regulation of apoptotic process | 0.009 | 1.52 | 1.65 | 27 |
|  | adenylate cyclase-activating G-protein coupled receptor signalling pathway | 0.009 | 0.96 | 1.95 | 17 |
|  | transmembrane transport | 0.010 | 2.14 | 1.50 | 38 |
|  | negative regulation of endopeptidase activity | 0.010 | 0.39 | 3.39 | 7 |
|  |  |  |  |  |  |
| KEGG | Cytokine-cytokine receptor interaction | 0.000 | 3.94 | 3.03 | 70 |
|  | Neuroactive ligand-receptor interaction | 0.000 | 3.21 | 1.91 | 57 |
|  | Cell cycle | 0.000 | 2.42 | 2.00 | 43 |
|  | DNA replication | 0.000 | 0.90 | 3.30 | 16 |
|  | Fanconi anemia pathway | 0.000 | 1.07 | 2.45 | 19 |
|  | Calcium signalling pathway | 0.001 | 2.59 | 1.60 | 46 |
|  | ECM-receptor interaction | 0.001 | 1.24 | 2.06 | 22 |
|  | Necroptosis | 0.002 | 1.52 | 1.85 | 27 |
|  | Cell adhesion molecules | 0.002 | 1.46 | 1.87 | 26 |
|  | Influenza A | 0.003 | 1.63 | 1.74 | 29 |
|  | NOD-like receptor signalling pathway | 0.005 | 1.69 | 1.66 | 30 |
|  | p53 signalling pathway | 0.009 | 1.07 | 1.86 | 19 |

**Supplementary Table 4.** CARLPS vs CARCTR. Showing all genes involved in immunomodulatory pathways (*P* < 0.01) enriched in the spleen of broiler embryos at d14 post-hatching after in ovo delivery of carvacrol at E17.5 and receiving lipopolysaccharide (LPS) at d7 and d14 post hatching. Input differentially expressed genes (DEG) were identified as FDR <0.05 and -0.5> logFC > 0.5. Databases used are Gene Ontology (GO) and Kyoto Encyclopedia of Genes and Genomes (KEGG).

|  |  |  |  |  |  | DEG enriching the pathways | |
| --- | --- | --- | --- | --- | --- | --- | --- |
| Database | Pathway Terms | *P*-value | % enriched | Fold enrichment | Number of DEG | Upregulated by LPS | Downregulated by LPS |
| GO | Immune response | 0.000 | 3.21 | 3.27 | 57 | AX31, ACKR4, B2M, BDKRB1, BF1, BF2, C8B, CCL19, CCL20, CCL4, CCL5, CCR2, CCR7, CD274, CD40, CSF3, CTLA4, CTSV, CX3CL1, CXCL13L2, CXCL13L3, CXCR1, CXCR4, FAS, FTH1, HHLA2, IL10, IL15, IL18, IL1B, IL6, IL8L1, IL8L2, NFIL3, TGFBR3, TLR15, TLR1B, TLR3, TLR5, TNFSF10, TNFSF15, TNFSF8 | APLNR, BLB1, CCL17, CCR6, CCR8L, CX3CR1, CXCL12, DMA, DMB2, GPR15, LIF, LY86, MHCY15, TNFSF13B, XCR1 |
|  | Inflammatory response | 0.000 | 2.25 | 2.86 | 40 | ADAM8, BCL6, BDKRB1, CCL19, CCL20, CCL4, CCL5, CCR2, CCR7, CD40, CD44, CX3CL1, CXCL13, CXCL13L2, CXCL13L3, EXFABP, IL18, IL1B, IL1R2, IL1RAP, IL8L1, IL8L2, MAPKAPK2, NFKB2, PTAFR, PTGER2, PTGFR, TLR15, TLR1B, TLR3, TLR5, TNFRSF4 | CCL17, GPR1, HMGB1, IL1RAPL1, KIT, LY86, RARRES2, WNT5A |
|  | Neutrophil chemotaxis | 0.000 | 0.84 | 4.50 | 15 | BDKRB1, BSG, CCL19, CCL20, CCL4, CCL5, CX3CL1, CXCL13, CXCL13L2, CXCL13L3, CXCR1, IL8L1, IL8L2, LGALS3 | CCL17 |
|  | Chemokine-mediated signalling pathway | 0.000 | 0.68 | 5.40 | 12 | CCL19, CCL20, CCL4, CCL5, CX3CL1, CXCL13, CXCL13L2, CXCL13L3, IL8L1, IL8L2 | CCL17, CXCL12 |
|  | Cellular response to lipopolysaccharide | 0.000 | 0.96 | 3.15 | 17 | CD274, CMPK2, CXCL13, CXCL13L2, CXCL13L3, HHLA2, IL10, IL1B, IL6, IL8L1, IL8L2, LY75, LY96, TNIP3, ZC3H12A | HMGB2, WNT5A |
|  | Innate immune response | 0.000 | 1.69 | 2.15 | 30 | C1R, C8B, DHX58, EXFABP, FCER1G, FOSL2, GBP, IFIH1, LY96, NFKB2, NMI, NOD1, OASL, PTX3, RIPK2, RSAD2, SAMHD1, TIFA, TLR15, TLR1B, TLR3, TLR5, TMEM173, TRIM35 | HCK, HMGB1, HMGB2, LY86, PTK6, RARRES2 |
|  | Response to lipopolysaccharide | 0.000 | 0.68 | 3.44 | 12 | CCR7, CEBPB, EXFABP, FOSL2, GCH1, IFNAR1, MAPKAPK2, MT4, PTGER2, PTGFR, TRIB1 | HMGB2 |
|  | Lymphocyte chemotaxis | 0.001 | 0.39 | 4.90 | 7 | CCL19, CCL20, CCL4, CCL5, CX3CL1, IL8L2 | CCL17 |
|  | Cytokine-mediated signalling pathway | 0.002 | 0.90 | 2.34 | 16 | CAV1, CSF2RA, GHR, IL13RA2, IL1B, IL21R, IL22RA1, IL7R, OSMR, RIPK2, SOCS1, STAT5A | CNTFR, FLT3, MPL, PRLR |
|  | Monocyte chemotaxis | 0.002 | 0.45 | 3.88 | 8 | CCL19, CCL20, CCL4, CCL5, CX3CL1, IL8L2, LGALS3 | CCL17 |
|  | Positive regulation of tumor necrosis factor production | 0.002 | 0.45 | 3.88 | 8 | HSPB1, IFIH1, IL6, MAPKAPK2, NOD1, RIPK2, TNFRSF8 | WNT5A |
|  | Defense response to virus | 0.002 | 0.68 | 2.70 | 12 | BCL2L1, CD40, DHX58, IFIH1, IFIT5, IL6, MLKL, MX1, OASL, RSAD2, SAMHD1, TMEM173 |  |
|  | Chemotaxis | 0.002 | 0.68 | 2.70 | 12 | ACKR2, ACKR4, CCR2, CCR7, CXCR1, CXCR4, PROK2, PTAFR | CCR6, CCR8L, CX3CR1, XCR1 |
|  | Cellular response to interleukin-1 | 0.006 | 0.39 | 3.68 | 7 | CCL19, CCL20, CCL4, CCL5, CD40, CX3CL1 | CCL17 |
|  | Positive regulation of interleukin-6 production | 0.006 | 0.39 | 3.68 | 7 | ADORA2B, IFIH1, IL1RAP, IL6, NOD1, RIPK2 | WNT5A |
|  | Acute-phase response | 0.007 | 0.28 | 5.25 | 5 | EXFABP, FN1, IL1B, IL6, SAA |  |
|  | Negative chemotaxis | 0.008 | 0.51 | 2.84 | 9 | SEMA4G, SEMA6C, SEMA7A | SEMA3G, SEMA4D, SEMA6A, SLIT1, SLIT2, SLIT3 |
|  | Negative regulation of apoptotic process | 0.009 | 1.52 | 1.65 | 27 | BCL6, BIRC2, CAPN3, CITED2, EXFABP, FAS, GAPDH, HSP90B1, HSPA5, HSPB1, IL6, OXSR1, PIM1, PIM3, PLK3, PROK2, PTGFR, SPHK1, STAT5A, TNFRSF18 | ANKRD1, BIRC5, FHL2, IGF1, MAD2L1, PLK1, WNT5A |
|  |  |  |  |  |  |  |  |
| KEGG | Cytokine-cytokine receptor interaction | 0.000 | 3.94 | 3.03 | 70 | ACKR4, BMP5, BMP7, CCL19, CCL20, CCL4, CCL5, CCR2, CCR7, CD40, CSF2RA, CSF3, CX3CL1, CXCL13, CXCL13L2, CXCL13L3, CXCR1, CXCR4, EDA2R, FAS, GHR, IFNAR1, IL10, IL10RA, IL10RB, IL13RA2, IL15, IL18, IL18RAP, IL1B, IL1R1, IL1R2, IL1RAP, IL1RL1, IL20RA, IL21R, IL22, IL6, IL7R, IL8L1, IL8L2, INHBB, OSMR, TNFRSF10B, TNFRSF11A, TNFRSF11B, TNFRSF18, TNFRSF25, TNFRSF4, TNFRSF6B, TNFRSF8, TNFRSF9, TNFSF10, TNFSF15, TNFSF8 | CCL17, CCR6, CCR8L, CNTFR, CX3CR1, CXCL12, GDF11, GDF9, LIF, MPL, NGFR, PRLR, TGFB3, TNFSF13B, XCR1 |
|  | Influenza A | 0.003 | 1.63 | 1.74 | 29 | BID, CASP1, CCL5, EIF2AK2, FAS, FDPS, IFIH1, IFNAR1, IKBKE, IL18, IL1B, IL6, IL8L1, IL8L2, MX1, PLG, RSAD2, SOCS3, STAT1, STAT2, TLR3, TNFRSF10B, TNFSF10, VDAC1 | BLB1, DMA, DMB2, KPNA2, SLC25A4 |
|  | NOD-like receptor signalling pathway | 0.005 | 1.69 | 1.66 | 30 | BCL2L1, BIRC2, CASP1, CCL5, CTSB, GBP, HSP90AA1, IFNAR1, IKBKE, IL18, IL1B, IL6, IL8L1, IL8L2, JUN, MAP1LC3A, MAP1LC3C, MAPK11, NOD1, PSTPIP1, RIPK2, STAT1, STAT2, TMEM173, TNFAIP3, TRAF2, TRPV2, VDAC1 | MAPK13, PLCB4 |
|  | p53 signalling pathway | 0.009 | 1.07 | 1.86 | 19 | BCL2L1, BID, CCNG1, CDKN1A, FAS, RPRM, SESN2, SHISA5, STEAP3, TNFRSF10B | ADGRB1, ATR, CCNB1, CCNE2, CDK1, CDK2, CHEK1, IGF1, RRM2 |

ACKR2 = atypical chemokine receptor 2; ACKR4 = atypical chemokine receptor 4; ADAM8 = ADAM metallopeptidase domain 8; ADGRB1 = adhesion G protein‑coupled receptor B1; ADORA2B = adenosine A2b receptor; ANKRD1 = ankyrin repeat domain 1; APLNR = apelin receptor; ATR = ATR serine/threonine kinase; B2M = beta‑2‑microglobulin; BCL2L1 = BCL2 like 1; BCL6 = B‑cell CLL/lymphoma 6; BDKRB1 = bradykinin receptor B1; BF1 = major histocompatibility complex B, class I heavy chain BF1; BF2 = major histocompatibility complex B, class I heavy chain BF2; BID = BH3 interacting domain death agonist; BIRC2 = baculoviral IAP repeat containing 2; BIRC5 = baculoviral IAP repeat containing 5; BLB1 = major histocompatibility complex Y, class II beta BLB1; BMP5 = bone morphogenetic protein 5; BMP7 = bone morphogenetic protein 7; BSG = basigin; C1R = complement C1r; C8B = complement C8 beta chain; CAPN3 = calpain 3; CASP1 = caspase 1; CAV1 = caveolin 1; CCL17 = C‑C motif chemokine ligand 17; CCL19 = C‑C motif chemokine ligand 19; CCL20 = C‑C motif chemokine ligand 20; CCL4 = C‑C motif chemokine ligand 4; CCL5 = C‑C motif chemokine ligand 5; CCNB1 = cyclin B1; CCNE2 = cyclin E2; CCNG1 = cyclin G1; CCR2 = C‑C motif chemokine receptor 2; CCR6 = C‑C motif chemokine receptor 6; CCR7 = C‑C motif chemokine receptor 7; CCR8L = C‑C chemokine receptor 8‑like; CD274 = CD274 molecule; CD40 = CD40 molecule; CD44 = CD44 molecule; CDK1 = cyclin‑dependent kinase 1; CDK2 = cyclin‑dependent kinase 2; CDKN1A = cyclin‑dependent kinase inhibitor 1A; CEBPB = CCAAT/enhancer‑binding protein beta; CHEK1 = checkpoint kinase 1; CITED2 = Cbp/p300 interacting transactivator with Glu/Asp‑rich C‑terminal domain 2; CMPK2 = cytidine/uridine monophosphate kinase 2; CNTFR = ciliary neurotrophic factor receptor; CSF2RA = colony‑stimulating factor 2 receptor alpha subunit; CSF3 = colony‑stimulating factor 3; CTLA4 = cytotoxic T‑lymphocyte–associated protein 4; CTSB = cathepsin B; CTSV = cathepsin V; CX3CL1 = C‑X3‑C motif chemokine ligand 1; CX3CR1 = C‑X3‑C motif chemokine receptor 1; CXCL12 = C‑X‑C motif chemokine ligand 12; CXCL13 = C‑X‑C motif chemokine ligand 13; CXCL13L2 = C‑X‑C motif chemokine ligand 13‑like 2; CXCL13L3 = C‑X‑C motif chemokine ligand 13‑like 3; CXCR1 = C‑X‑C motif chemokine receptor 1; CXCR4 = C‑X‑C motif chemokine receptor 4; DHX58 = DExH‑box helicase 58; DMA = major histocompatibility complex B, class II alpha chain DMA; DMB2 = major histocompatibility complex B, class II beta chain DMB2; EDA2R = ectodysplasin A2 receptor; EIF2AK2 = eukaryotic translation initiation factor 2‑alpha kinase 2; EXFABP = extracellular fatty acid‑binding protein; FAS = Fas cell surface death receptor; FCER1G = Fc fragment of IgE receptor gamma; FDPS = farnesyl diphosphate synthase; FHL2 = four and a half LIM domains 2; FLT3 = Fms‑related tyrosine kinase 3; FN1 = fibronectin 1; FOSL2 = FOS‑like 2; FTH1 = ferritin heavy chain 1; GAPDH = glyceraldehyde‑3‑phosphate dehydrogenase; GBP = guanylate‑binding protein; GCH1 = GTP cyclohydrolase 1; GDF11 = growth differentiation factor 11; GDF9 = growth differentiation factor 9; GHR = growth hormone receptor; GPR1 = G protein‑coupled receptor 1; GPR15 = G protein‑coupled receptor 15; HCK = HCK proto‑oncogene, Src family tyrosine kinase; HHLA2 = HHLA2, member of B7 family; HMGB1 = high mobility group box 1; HMGB2 = high mobility group box 2; HSP90AA1 = heat shock protein 90 alpha family class A member 1; HSP90B1 = heat shock protein 90 beta family member 1; HSPA5 = heat shock 70 kDa protein 5; HSPB1 = heat shock protein family B member 1; IFIH1 = interferon‑induced with helicase C domain 1; IFIT5 = interferon‑induced protein with tetratricopeptide repeats 5; IFNAR1 = interferon alpha/beta receptor subunit 1; IGF1 = insulin‑like growth factor 1; IKBKE = inhibitor of nuclear factor kappa‑B kinase subunit epsilon; IL10 = interleukin 10; IL10RA = interleukin 10 receptor subunit alpha; IL10RB = interleukin 10 receptor subunit beta; IL13RA2 = interleukin 13 receptor subunit alpha 2; IL15 = interleukin 15; IL18 = interleukin 18; IL18RAP = interleukin 18 receptor accessory protein; IL1B = interleukin 1β; IL1R1 = interleukin 1 receptor type 1; IL1R2 = interleukin 1 receptor type 2; IL1RAP = interleukin 1 receptor accessory protein; IL1RAPL1 = interleukin 1 receptor accessory protein‑like 1; IL1RL1 = interleukin 1 receptor‑like 1; IL20RA = interleukin 20 receptor subunit alpha; IL21R = interleukin 21 receptor; IL22 = interleukin 22; IL22RA1 = interleukin 22 receptor subunit alpha 1; IL6 = interleukin 6; IL7R = interleukin 7 receptor; IL8L1 = interleukin 8‑like 1; IL8L2 = interleukin 8‑like 2; INHBB = inhibin beta B subunit; JUN = Jun proto‑oncogene, AP‑1 transcription factor subunit; KIT = KIT proto‑oncogene, receptor tyrosine kinase; KPNA2 = karyopherin subunit alpha 2; LGALS3 = galectin 3; LIF = leukemia inhibitory factor; LY75 = lymphocyte antigen 75; LY86 = lymphocyte antigen 86; LY96 = lymphocyte antigen 96; MAD2L1 = mitotic arrest deficient 2‑like 1; MAP1LC3A = microtubule‑associated protein 1 light chain 3 alpha; MAP1LC3C = microtubule‑associated protein 1 light chain 3 gamma; MAPK11 = mitogen‑activated protein kinase 11; MAPK13 = mitogen‑activated protein kinase 13; MAPKAPK2 = MAPK‑activated protein kinase 2; MHCY15 = major histocompatibility complex Y, class I heavy chain 15; MLKL = mixed lineage kinase domain‑like pseudokinase; MPL = MPL proto‑oncogene, thrombopoietin receptor; MT4 = metallothionein 4; MX1 = myxovirus resistance 1; NFIL3 = nuclear factor, interleukin‑3 regulated; NFKB2 = nuclear factor kappa‑B subunit 2; NGFR = nerve growth factor receptor; NMI = N‑myc and STAT interactor; NOD1 = nucleotide‑binding oligomerization domain‑containing 1; OASL = 2′‑5′‑oligoadenylate synthetase‑like; OSMR = oncostatin M receptor; OXSR1 = oxidative stress responsive 1; PIM1 = Pim‑1 proto‑oncogene, serine/threonine kinase; PIM3 = Pim‑3 oncogene; PLCB4 = phospholipase C beta 4; PLG = plasminogen; PLK1 = polo‑like kinase 1; PLK3 = polo‑like kinase 3; PRLR = prolactin receptor; PROK2 = prokineticin 2; PSTPIP1 = proline‑serine‑threonine phosphatase‑interacting protein 1; PTAFR = platelet‑activating factor receptor; PTGER2 = prostaglandin E receptor 2; PTGFR = prostaglandin F receptor; PTK6 = protein tyrosine kinase 6; PTX3 = pentraxin 3; RARRES2 = retinoic acid receptor responder 2; RIPK2 = receptor‑interacting serine/threonine kinase 2; RPRM = reprimo, TP53‑dependent G2 arrest mediator homolog; RRM2 = ribonucleotide reductase regulatory subunit M2; RSAD2 = radical S‑adenosyl methionine domain‑containing 2; SAA = serum amyloid A; SAMHD1 = SAM and HD domain‑containing deoxynucleoside triphosphate triphosphohydrolase 1; SEMA3G = semaphorin 3G; SEMA4D = semaphorin 4D; SEMA4G = semaphorin 4G; SEMA6A = semaphorin 6A; SEMA6C = semaphorin 6C; SEMA7A = semaphorin 7A; SESN2 = sestrin 2; SHISA5 = shisa family member 5; SLC25A4 = solute carrier family 25 member 4; SLIT1 = slit guidance ligand 1; SLIT2 = slit guidance ligand 2; SLIT3 = slit guidance ligand 3; SOCS1 = suppressor of cytokine signaling 1; SOCS3 = suppressor of cytokine signaling 3; SPHK1 = sphingosine kinase 1; STAT1 = signal transducer and activator of transcription 1; STAT2 = signal transducer and activator of transcription 2; STAT5A = signal transducer and activator of transcription 5A; STEAP3 = STEAP3 metalloreductase; TGFB3 = transforming growth factor beta 3; TGFBR3 = transforming growth factor beta receptor 3; TIFA = TRAF‑interacting protein with forkhead‑associated domain; TLR15 = Toll‑like receptor 15; TLR1B = Toll‑like receptor 1 family member B; TLR3 = Toll‑like receptor 3; TLR5 = Toll‑like receptor 5; TMEM173 = transmembrane protein 173; TNFAIP3 = TNF alpha‑induced protein 3; TNFRSF10B = tumor necrosis factor receptor superfamily member 10B; TNFRSF11A = tumor necrosis factor receptor superfamily member 11A; TNFRSF11B = tumor necrosis factor receptor superfamily member 11B; TNFRSF18 = tumor necrosis factor receptor superfamily member 18; TNFRSF25 = tumor necrosis factor receptor superfamily member 25; TNFRSF4 = tumor necrosis factor receptor superfamily member 4; TNFRSF6B = tumor necrosis factor receptor superfamily member 6B; TNFRSF8 = tumor necrosis factor receptor superfamily member 8; TNFRSF9 = tumor necrosis factor receptor superfamily member 9; TNFSF10 = tumor necrosis factor superfamily member 10; TNFSF13B = tumor necrosis factor superfamily member 13B; TNFSF15 = tumor necrosis factor superfamily member 15; TNFSF8 = tumor necrosis factor superfamily member 8; TNIP3 = TNFAIP3‑interacting protein 3; TRAF2 = TNF receptor‑associated factor 2; TRIB1 = tribbles pseudokinase 1; TRIM35 = tripartite motif‑containing 35; TRPV2 = transient receptor potential cation channel subfamily V member 2; VDAC1 = voltage‑dependent anion channel 1; WNT5A = Wnt family member 5A; XCR1 = X‑C motif chemokine receptor 1; ZC3H12A = zinc finger CCCH‑type containing 12A.

**Supplementary Table 5.** CARLPS vs CTRLPS. All physiological pathways (*P* < 0.01) enriched in the spleen of broiler embryos at d14 post-hatching after in ovo delivery of carvacrol at E17.5, compared to in ovo delivery of saline, both receiving lipopolysaccharide (LPS) challenges at d7 and d14 post hatching. Input differentially expressed genes (DEG) were identified as *P*-value < 0.05, -0.5> logFC > 0.5. Databases used are Gene Ontology (GO) and Kyoto Encyclopedia of Genes and Genomes (KEGG).

| Database | Term | *P*-value | % enriched | Fold enrichment | Number of DEG |
| --- | --- | --- | --- | --- | --- |
| GO | defense response to virus | 0.000 | 1.85 | 7.69 | 13 |
|  | immune response | 0.000 | 3.13 | 3.31 | 22 |
|  | cellular response to interleukin-1 | 0.000 | 0.99 | 9.66 | 7 |
|  | positive regulation of ERK1 and ERK2 cascade | 0.000 | 1.70 | 3.68 | 12 |
|  | cell adhesion | 0.000 | 3.13 | 2.32 | 22 |
|  | cellular response to tumor necrosis factor | 0.001 | 0.99 | 5.52 | 7 |
|  | lymphocyte chemotaxis | 0.001 | 0.71 | 9.20 | 5 |
|  | G-protein coupled receptor signalling pathway | 0.001 | 2.98 | 2.19 | 21 |
|  | extracellular matrix organization | 0.002 | 1.70 | 3.06 | 12 |
|  | regulation of vasoconstriction | 0.004 | 0.57 | 11.04 | 4 |
|  | cellular response to interferon-gamma | 0.004 | 0.85 | 5.23 | 6 |
|  | negative regulation of gene expression | 0.005 | 1.42 | 3.01 | 10 |
|  | axon guidance | 0.005 | 1.85 | 2.50 | 13 |
|  | cholesterol homeostasis | 0.006 | 0.85 | 4.97 | 6 |
|  | regulation of cytosolic calcium ion concentration | 0.006 | 0.71 | 6.37 | 5 |
|  | monocyte chemotaxis | 0.006 | 0.71 | 6.37 | 5 |
|  | transmembrane transport | 0.008 | 2.70 | 1.97 | 19 |
|  | cell differentiation | 0.008 | 3.27 | 1.81 | 23 |
|  | DNA unwinding involved in DNA replication | 0.008 | 0.71 | 5.92 | 5 |
|  | chemokine-mediated signalling pathway | 0.008 | 0.71 | 5.92 | 5 |
|  | regulation of cardiac muscle contraction | 0.010 | 0.57 | 8.28 | 4 |
|  |  |  |  |  |  |
| KEGG | Neuroactive ligand-receptor interaction | 0.000 | 4.69 | 3.06 | 33 |
|  | Cytokine-cytokine receptor interaction | 0.000 | 3.84 | 3.22 | 27 |
|  | Calcium signalling pathway | 0.000 | 4.12 | 2.78 | 29 |
|  | Vascular smooth muscle contraction | 0.000 | 2.41 | 3.25 | 17 |
|  | Arachidonic acid metabolism | 0.004 | 0.99 | 4.42 | 7 |
|  | Adrenergic signalling in cardiomyocytes | 0.004 | 2.13 | 2.34 | 15 |
|  | Cytosolic DNA-sensing pathway | 0.010 | 1.14 | 3.24 | 8 |
